# Optical Fiber Displacement Sensor Based on Microwave Photonics Interferometry

**DOI:** 10.3390/s18113702

**Published:** 2018-10-31

**Authors:** Hao Dong, Shicheng Liu, Liming Yang, Jiangbo Peng, Keming Cheng

**Affiliations:** 1 College of Aerospace Engineering, Nanjing University of Aeronautics and Astronautics, Nanjing 210016, China; liushicheng@nuaa.edu.cn (S.L.); cheng.km@nuaa.edu.cn (K.C.); 2 Department of Mechanical Engineering, National University of Singapore, Singapore 119260, Singapore; mpeylim@nus.edu.sg; 3 Institute of Opt-Electronics, Harbin Institute of Technology, Harbin 150080, China; pengjiangbo_2004@126.com

**Keywords:** displacement sensor, microwave photonics, position sensor, interferometry

## Abstract

An optical fiber displacement sensor based on the microwave photonics interferometric (MWPI) method is proposed and experimented, which provides an ideal solution for large range displacement measurement with high resolution. The sensor used a Michelson microwave photonics interferometer to sense the displacement with one sensing arm and a length-adjusted reference arm. The displacement variation would change the period of the microwave response function of the interferometer. According to the principle that the phase difference in one free spectral range (FSR) of the microwave response function is 360°, the displacement can be retrieved by the microwave response function by means of a vector network analyzer (VNA). A programmable path-switching true time delay line was used in the reference arm to decrease the microwave bandwidth. The measurement results show that the displacement sensing range is larger than 3 m and the measurement resolution is 31 μm. Finally, the measurement stability is tested, and the factors affecting the measurement resolution of this method and the main source of errors are investigated in detail.

## 1. Introduction

Displacement measurements are fundamentally important in industrial applications, and the displacement sensors are gaining more important attention in the increasing automated industry [1]. Kinds of displacement sensors have been proposed, including capacitive displacement sensors [2], eddy-current sensors [3], linear variable displacement transformer (LVDTs) [4], linear optical grating [5], laser interferometers [6], and magneto-strictive displacement sensors [7]. The displacement measurement resolution of these sensors is as high as a nanometer level with hundreds of millimeters, which plays a key role in automation control application. Over the past few decades, optical fiber displacement sensors have been developed with considerable attention to their advantage of lightweight, high resolution immunity to electromagnetic interference, remote sensing ability, etc.; such sensors have been widely used in slope sliding monitoring, dam deformation, foundation settlement, steel corrosion, earthquakes and so on [8].

Various types of optical fiber sensing techniques have been developed for displacement measurement, including Fabry-Perot interferometers [9,10], multimode interference, fiber Bragg gratings [11,12], surface plasmon resonance [13], white light interferometers (WLI) [14,15], and air-cavity methods [16]. Most of these methods rely on the optical interference with high measurement resolution. However, such methods are usually unstable. In recent years, the microwave photonics (MWP) optical fiber sensing technology has raised increasing interest. The MWP technique combines the advantages of both the microwave and photonics techniques, which enable this technique to sense several parameters in a variety of sensing applications. This technique down-coverts the optical interference to the RF region to sense the measured parameters, which is far more stable and easier to control with fast sensing speed and low cost. Such a technique can achieve a larger dynamic range (DR) with a high measurement resolution. There are several types of optical fiber sensors based on the microwave photonic (MWP) technology. One method uses the opto-electric oscillator (OEO) to sense the displacement and other parameters [17]. The displacement of the object to be tested produces a shift of the oscillator frequency of the output microwave signal from the OEO. The microwave frequency from the OEO has a high spectral purity and can be measured by the spectrum analyzer directly with high resolution over a large measurement range [18]. MWP interferometry using a low coherence white light source has also been developed to be used in optical path difference sensing [19]. This method uses microwave interference to demodulate the tested parameters. In addition, the MWP filter can also be used to measure the distance and position with a large range [20]. Table 1 summarizes the characteristics of the above displacement sensors.

In this paper, a microwave photonics interferometric (MWPI) technique for a non-contact optical fiber displacement sensor was presented with large displacement measurement and high resolution. In addition, this sensor could measure the displacement and position simultaneously. The method adopted a microwave photonics optical fiber Michelson interferometer (MI) and used the microwave interference fringes to demodulate the displacement. A vector network analyzer (VNA) was used to measure and record the microwave response function (S21 parameter) of the sensing system. The displacement of the object to be tested in the sensing arm in the MI would change the phase of the interferometer, which would change the period of the microwave response function out of the interferometer. According to the principle that the phase difference in one free spectral rang (FSR) of the microwave response function is 360°, the displacement can be retrieved by the FSR from the recorded S21 parameters with a simple operation, rapid measurement time and high signal-to-noise ratio. We adopted a programmable path-switching true time delay line in the reference arm to decrease the microwave bandwidth of the devices used in the sensor, which can enlarge the application scope and decrease the cost of the sensor. The performance of the sensor was tested, and the experiment results showed that the measurement resolution of the sensor is 31 μm and the measurement range is larger than 3 m.

## 2. Materials and Methods

**Figure 1:**
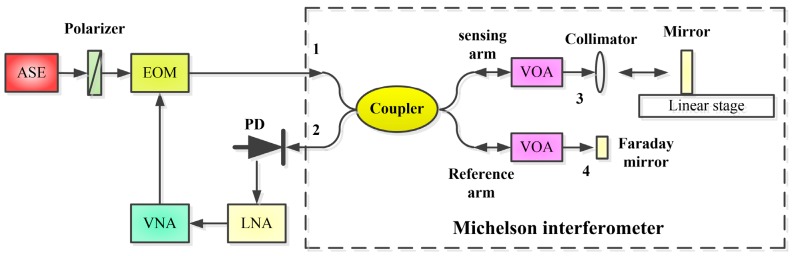
Schematic of the displacement sensor based on MWPI.

The schematic diagram of the optical fiber sensor based on the MWPI using a microwave photonics optical fiber MI is shown in Figure 1.

Linear polarized light from a broadband amplified spontaneous emission (ASE) light is injected into an electro-optic intensity modulator (EOM). The light is amplitude-modulated by an RF signal from a VNA, and the electro-optic intensity modulator (EOM) is biased at quadrature. The amplitude-modulated light from the EOM is launched into an optical fiber MI. The output light from Port 2 is received by a photodiode (PD), and the detected RF signal from the PD is amplified by a low noise amplifier (LNA). The amplified RF signal from the LNA is received by the VNA. The displacement variation of the mirror in the sensing arm will cause the phase shift of the microwave interference fringes at the output of the PD. The voltage injected into the EOM contains DC bias and the RF signal, which can be expressed as

(1) $$V_{in}(t)=V_{dc}+V_{RF}\cos(2\pi f_{m}t)$$

where $V_{dc}$ and $V_{RF}$ are the DC bias and RF signal voltage applied on the EOM, respectively, $f_{m}$ is the frequency of the RF signal. The optical intensity out of the EOM is

(2) $$I(t)=\xi I_{0}\cos^{2}\left(\varphi (t)\right)=\frac{1}{2}\xi I_{0}\left[1+\cos\left(\frac{\pi V_{dc}}{V_{\pi }}+\frac{\pi V_{RF}\cos2\pi f_{m}}{V_{\pi }}\right)\right]$$

where $\varphi (t)=\pi V_{dc}/V_{\pi }+\pi V_{RF}\cos2\pi f_{m}/V_{\pi }$ is the phase shift induced by the EOM, $V_{\pi }$ is the half-wave voltage of the EOM, ξ is the optical power loss factor of the EOM. The intensity-modulated light was injected into the MI. If the optical path from Port 1 to Port 3 is equal to that from Port 1 to Port 4, the two reflection lights from the reference and sensing arms can be expressed as

(3) $$I_{1}(t)=\frac{1}{4}\xi I_{0}\left[1+\cos\left(\frac{\pi V_{DC}}{V_{\pi }}+\frac{\pi V\cos2\pi f_{m}}{V_{\pi }}\right)\right]$$

(4) $$I_{2}(t)=\frac{1}{4}\xi I_{0}\left[1+\cos\left(\frac{\pi V_{DC}}{V_{\pi }}+\frac{\pi V\cos(2\pi f_{m}+\Delta \phi )}{V_{\pi }}\right)\right]$$

where Δϕ is the phase difference of the two arms in the MI and can be expressed as

(5) $$\Delta \phi =4\pi f_{m}L/c$$

where L is the distance between the scanning mirror and the output of the Port 3, c is the velocity of the light. The two lights were detected by the PD, and the RF signal out of the PD is

(6) $$I_{out}(t)=\xi R\rho J_{1}\left(\frac{\pi V_{RF}}{V_{\pi }}\right)\left[\sin\left(2\pi f_{m}+\Delta \phi \right)+\sin\left(2\pi f_{m}\right)\right]$$

where R is the load, ρ is the response of the PD, $J_{1}$ is the 1th-order Bessel function of the first kind. In the VNA, there is a local oscillator (LO) that can be expressed as

(7) $$V_{LO}(t)=V_{LO}\cos(2\pi f_{m}t)$$

The received RF signal from the PD is mixed with the LO signal by a mixer in the VNA, and the microwave response function of the sensor measured by the VNA can be expressed as

(8) $$H\left(f_{m}\right)\propto \left(1+\sin\left(\frac{4\pi f_{m}L}{c}\right)\right)$$

From Equation (8) we know that the output of the PD is characterized by period spectral, and the frequency difference in one period is named the free spectral rang (FSR). In one period, the phase difference is 3600, namely,

(9) $$\frac{4\pi f_{m}L}{c}=2\pi$$

The absolute position of the mirror from output end of Port 3 can be expressed as

(10) $$L=\frac{c}{2\Delta f_{m}}$$

where $\Delta f_{m}$ is the FSR of the output microwave response function. When the FSRs of the output microwave response function are $\Delta f_{m1}$ and $\Delta f_{m2}$ before and after a displacement has happened, the displacement of the mirror can be expressed as

(11) $$\Delta L=\frac{c}{2}\left(\frac{1}{\Delta f_{m1}}-\frac{1}{\Delta f_{m2}}\right)$$

## 3. Results

The experimental setup is shown in Figure 1. The light source was a pigtailed broadband amplified spontaneous emission (ASE), which emitted white light with a central wavelength of 1545 nm and with a spectral bandwidth of 42 nm. The output optical power of the ASE was 20 mW. The white light was polarized by an optical fiber polarizer and then amplitude-modulated by a RF signal from the output port of a vector network analyzer (VNA) using an electro-optic intensity modulator (EOM) that was biased at quadrature. The half-wave voltage of the EOM (EOspace, AX-OMKS-20-PFA-PFA-LV) was 3.6 V at 1 GHz with an insert loss of 3.1 dB and a bandwidth of 20 GHz, respectively. The bias of the EOM was controlled by a circuit module (YYLab, MCB1) at quadrature. The intensity-modulated light from the EOM was injected into one of the input ports of a 2 × 2 directional single-mode optical fiber coupler which was used as an MI. One of the output arms was the sensing arm that connected an optical fiber collimator and the output light from the collimator is incident on a scanning mirror directly. The reflection light from the mirror was coupled into the collimator again. The other output arm of the interferometer was a reference arm with a programmable optical delay line and the output end was connected to a Faraday mirror. The Faraday rotation mirror was used in the reference arm to eliminate the influence of polarization fluctuation on the performance of the sensor. There were variated optical attenuators (VOA) in the two output arms of the interferometer. The function of the two VOAs was to adjust the optical power of the two reflection lights to be equal. The two reflection lights from the two arms were received by a high-speed photodiode. The microwave interference fringes were formed by the high-speed photodiode. The microwave interference fringes signal from the PD was amplified by a low noise amplifier (LNA) and the amplified RF signal was injected into the VNA. The VNA was used to measure the microwave response function of the sensor, and the two ports of the VNA were connected to the EOM and the LNA.

In our measurement, the light intensity received by the PD was −0.4 dBm. In order to obtain a good measurement result, the gain of the LNA was chosen as 26 dB at 12 GHz. In such case, the measured maximum gain at 12 GHz was −22 dB.

The sensor was designed by using the optical fiber MI. In the sensing arm, the output end of the arm was an optical fiber collimator which was the sensing head of the sensor. The diameter of the lens in the collimator is 10 mm. The light from the collimator is directed toward the scanning mirror which served as the object to be tested. The waist diameter of the light beam out of the collimator is 3.6 mm and the divergence angle is 0.030. The back-coupling efficiency is about 70% when the distance from the mirror and the collimator is 3 m. The scanning mirror was mounted on a linear long travel motorized stage whose travel range is 3 m and the minimum achievable increase is 1 μm.

**Figure 2:**
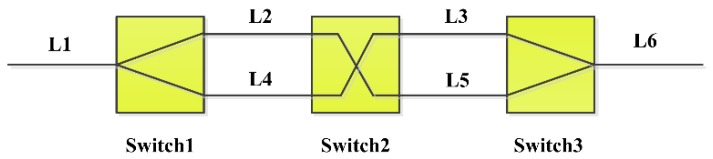
The setup of the programmable delay line.

In the MI, the optical path from the optical fiber coupler to the optical fiber collimator in the sensing arm is equal to the optical path from the optical fiber coupler to the optical fiber Faraday mirror. The optical path difference (OPD) of the MI is small when the mirror is near the optical fiber collimator. In such case, the FSR is so large that it needs high bandwidth microwave photonics devices, so a programmable optical delay line was used to enlarge the OPD when the mirror is near the optical fiber collimator to decrease the FSR. The programmable optical fiber delay line is a 2-bit programmable path-switching true time delay line using switching in and out of varying length of the optical fiber by an optical switch. The setup of the 2-bit programmable optical fiber delay line is shown in Figure 2.

The delay line includes two 1 × 2 MEMS optical switches and one 2 × 2 MEMS optical switch. The optical path of the minimum delay path is L1 + L2 + L3 + L6, which is equal to the optical path of the sensing arm with no scanning mirror. The 2-bit programmable delay line can provide four time-delays and the four delay intervals are available as shown in Table 2. Larger optical delay can be obtained by using larger bits in the delay line.

The total length of the minimum delay path is about 1 m; in that case, the temperature-induced optical delay variation is 0.4 ps when the temperature variation is 10 °C, which has no influence on the measurement. When the programmable optical delay line was used to decrease the microwave bandwidth, the Equation (11) can be rewritten as

(12) $$\Delta L=\frac{c}{2(n-1)}\left(\frac{1}{\Delta f_{m1}}-\frac{1}{\Delta f_{m2}}\right)$$

where n is the refractive index of the single mode optical fiber. The time delay range of the optical delay line is as large as several meters and the continuous delay line can hardly produce such large time delay.

**Figure 3:**
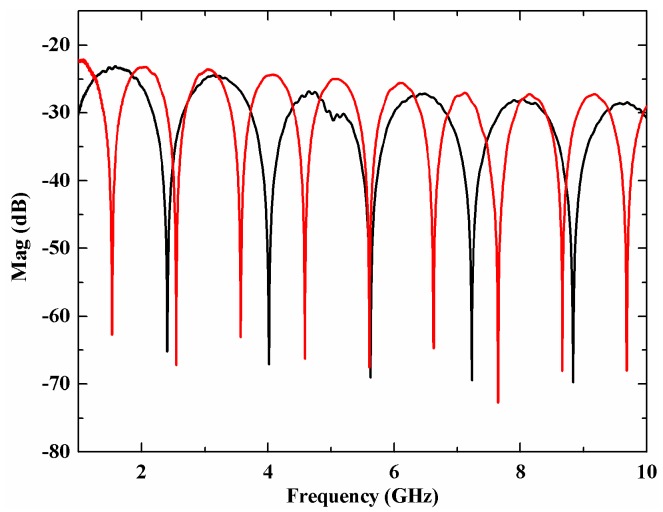
Measured S_21_ parameter before (black line) and after (red line) the displacement of the mirror.

To better understand the performance of the sensor, we moved the scanning mirror to cause a displacement of 540 mm and used the VNA to record the microwave response function of the sensor. Figure 3 shows the measured microwave response function of the sensor before and after the displacement of the scanning mirror by the VNA. The sweep range is from 1 GHz to 10 GHz, the sample point is 4000 and the IF bandwidth is 1 KHz. From Figure 3 we can find that the microwave response function shifts and the FSR changes when the position of the scanning mirror has a displacement. The FSR decreases when the scanning mirror is far away from the optical fiber collimator, which agrees well with the previous analysis. The optical loss in the sensing arm and the reference arm are different at different mirror positions; two VOAs were used to balance the optical power of the two arms to achieve a maximum visibility of the microwave interference patterns.

**Figure 4:**
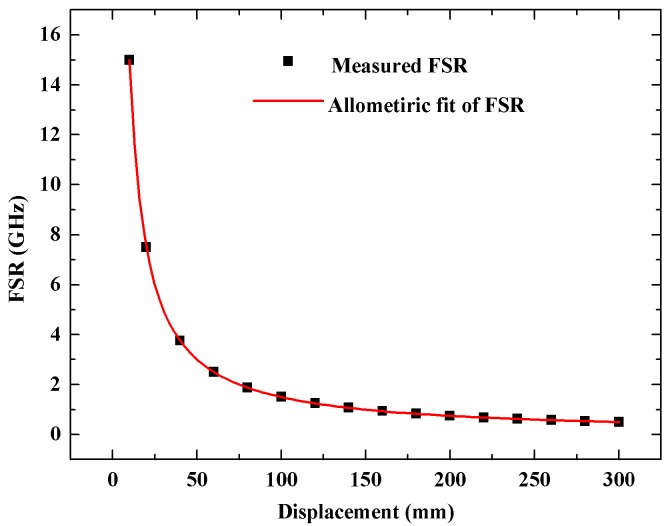
The relationship between the FSR and the displacement of the mirror.

The FSR from Figure 3 is 1.60712 GHz and 1.01807 GHz, and the calculated displacement is 540.029 mm, which agrees well with the displacement of the linear stage. We move the scanning mirror with several different displacements and record the microwave response function and then obtain the FSR at different displacements. Figure 4 shows the relationship between the displacement and the FSR and their line fitting results. The fitting expression is $\Delta f=149.78208L^{-0.99965}$, and the correlation coefficient is 0.99995. The fitting result agrees well with Equation (10) where the theoretical expression is $\Delta f=150L^{-1}$.

**Figure 5:**
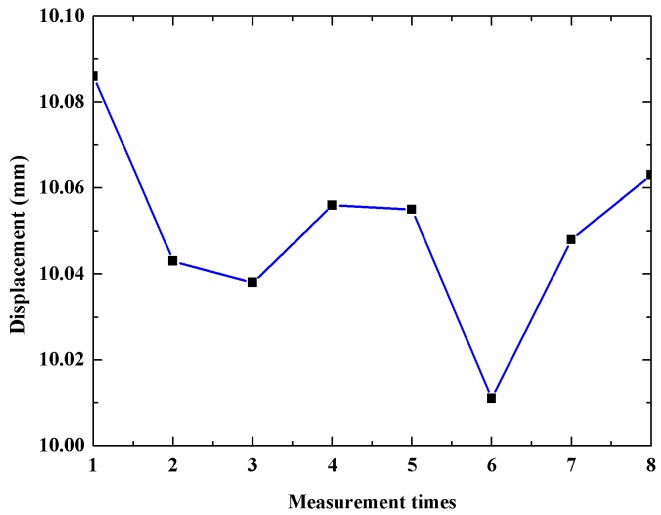
Measurement stability of the sensor.

To check the stability of the sensor, we move the scanning mirror with 10 mm ten times and use the proposed sensor to measure the displacement of the mirror. The measurement results are shown in Figure 5. The mean is 10.1052 mm and the standard deviation is 22 μm.

**Figure 6:**
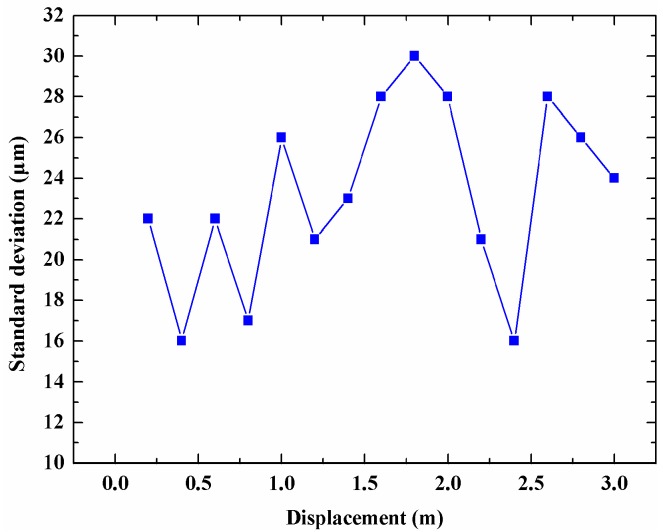
Measurement standard deviation at different displacements.

We also evaluate the displacement measurement standard deviation at different displacements from 200 mm to 3 m, and the measurement results are shown in Figure 6. The measurements at each displacement were conducted 10 times. As Figure 6 shows, the largest measurement standard deviation is 31 μm when the displacement is within 3 m. The minimum achievable increase of the motorized linear stage is 1 μm whose displacement error can be ignored in the measurement result, so the displacement measurement resolution of the proposed sensor was 31 μm.

It can be seen from Equation (10) that the measurement resolution of this MWPI was mainly limited by the FSR resolution obtained by the VNA. The measurement resolution of the displacement can be determined by the following equation

(13) $$\delta (L)=L\sqrt{\frac{{\left[\delta (\Delta f_{m})\right]}^{2}}{{\left(\Delta f_{m}\right)}^{2}}}$$

The frequency resolution of the VNA is as high as 1 Hz, which assures that the displacement measurement resolution is very high. According to Equation (13), the theoretical measurement resolution is 60 nm, which is more ideal than the experimental results. The higher error impacted on the measurement accuracy and resolution was caused by the FSR error.

The maximum displacement measured $L_{\max}$ was determined by two factors, including the maximum travel of the linear motorized stage and the divergence angle of the light beam out of the optical fiber collimator. The small divergence angle and beam diameter of the light beam assure high back-coupling efficiency when the travel range of the motorized stage is large. In our measurement, the maximum displacement measured is 3 m which is determined by the motorized stage. Larger displacement measurement can be achieved in our measurement for the divergence angle of the light beam is very small. As the displacement increases, the back-coupling efficiency is lower, and the optical power of the two arms should be adjusted by the two VOAs to assure a high interference visibility. The minimum measured $L_{\min}$ was also determined by the FSR resolution. There is a blind area when the scanning mirror is very close to the optical fiber collimator. In such case, the FSR is so large that it exceeds the frequency span of the VNA. In our measurement, the frequency span of the VNA is 100 KHz to 20 GHz. From Equation (10) we know that the minimum OPD of the MI is 7.9 mm, namely, the minimum distance between the scanning mirror and the optical fiber collimator is 7.9 mm when the optical path in the reference arm is L1 + L2 + L3 + L6.

## 4. Discussion

MWPI is a simple and powerful technology to measure the displacement rather than the current optical fiber sensor for large DR and high stability. In our measurement, the displacement measurement resolution is 31 μm which is determined by the FSR accuracy. The FSR accuracy is affected seriously by two factors that are the dynamic range (DR) and the noise floor of the microwave response function.

First, when the DR of the microwave response function is low, the valley of the interference fringes is flat and that can impact on the FSR accuracy. There are also two main factors that can impact the DR. One is the microwave interference visibility out of the PD. The interference visibility decreases when the optical power difference of the two arms is not zero. When the scanning mirror is far from the optical fiber collimator or the programmable delay line switches, the optical power will change. We used two VOAs to assure the optical power of the two arms were equal. The other one is the bias point of the EOM. When the bias point deviates the quadrature point, the dynamic range of the microwave interference signal will decrease and a large harmonic wave will appear in the signal.

**Figure 7:**
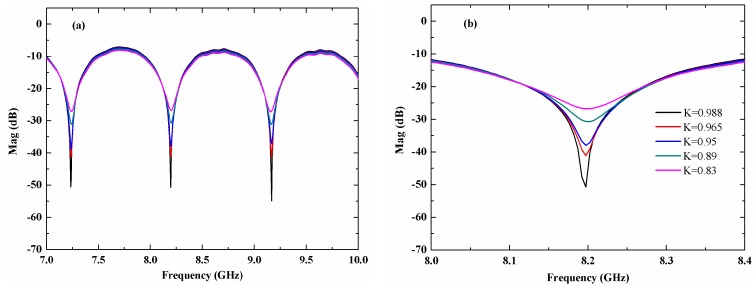
(**a**) Microwave response function at different dynamic ranges, (**b**) The detail of the valley point.

An inaccurate FSR result would be obtained if the interference visibility is low. Figure 7 shows the microwave response function with different DR by adjusting the VOA to change the interference visibility of the MI. As shown in Figure 7, the valley area of the microwave response function becomes much flatter as the interference visibility decreases. In Figure 7b, K represents the optical power ratio of the two arms.

Secondly, the noise floor of the sensor can also affect the FSR accuracy. The noise source of the RF signal includes thermal noise, shot noise of the PD, and relative intensity noise (RIN) of the light source. In our measurement, the RIN is the main noise source. In such a case, more accurate FSR can be easily obtained by measuring the microwave response function several times and averaging the measured data. Decreasing the IF bandwidth of the VNA and using a DFB light source with low RIN can improve the SNR of the microwave interference signal.

The fiber length in the two arms was short, and the fiber was wound to form a ring with a suitable bending radius. Such bending had no impact on the measurement resolution of the sensor if the bending optical fiber was in a steady state and the optical losses of the two arms had no change with bending. We also tested the performance of the sensor when the bending condition of the optical fiber was changing dynamically. The measurement resolution changed largely, which means the dynamic change of the optical fiber condition of the two arms had a serious influence on the performance of the sensor.

The measurement method is based on a microwave response function measurement with a VNA, whose signal-to-noise ratio can be at least 90 dB among 100 KHz to 20 GHz, and the measurement time is about 5 s when the sample point is 4000 and the IF bandwidth is 1 KHz. Under such circumstances, the temperature-induced measurement error is too small to be ignored. By using a programmable path-switching true time delay line, lower bandwidth frequency response devices can be used in our measurement to achieve large displacement measurement and high resolution. The displacement measurement range is determined by the travel range of the linear motorized stage. In practical application, the displacement measurement range is determined by the back-coupling efficiency of the optical fiber collimator and the signal noise ratio of the sensor. The sensing head can be installed easily and the sensor can work when the output light from the sensing head illuminates the surface of the object to be tested with high reflectivity. If the divergence angle of the light out of the optical fiber collimator is so small that it assures the back-coupling efficiency is large, the measurement range of the proposed sensor can reach as high as several kilometers. When the measurement range is as large as hundred meters, the measurement resolution will decrease. In such a case, the programmable path-switching true time delay line can be used to decrease the OPD of the MI to improve the measurement resolution. In addition, the absolute position of the scanning mirror from the output end of the optical fiber collimator can also be obtained by Equation (10). Therefore, this sensor can measure the displacement and absolute position or distance simultaneously. Based on these performances, the proposed sensor is especially suitable in non-contact, remote displacement sensing with a large-scale range.

## 5. Conclusions

In summary, we proposed an MWPI method for a non-contact optical fiber displacement sensor with large dynamic range and high resolution. The sensor is fabricated by an optical fiber MI and the microwave response parameter was measured to calculate the displacement parameter. One arm of the interferometer is the sensing arm and the reference arm is a programmable path-switching true time delay line. The displacement variation of the object to be tested would change the phase of the interferometer, which will change the FSR of the microwave response function out of the interferometer. According to the principle that the phase difference in one FSR of the microwave response function is 360°, the displacement can be retrieved by the FSR calculated from the recorded S21 parameters. A programmable path-switching true time delay line was used in the reference arm to decrease the frequency response bandwidth of the devices and instruments. The measurement results show that the measurement resolution is 31 μm.

The method has the merits of high signal-to-noise ratio and rapid measurement time, and it is insensitive to environment fluctuation. The displacement can be obtained by a simple measurement process with large DR and high resolution. The main factors which limit the measurement accuracy are the interference visibility of the MI, the bias point of the EOM and the RIN of the light source.

## Figures and Tables

**Table 1:** Summary of the characteristics of the major types of displacement sensors.

Sensor Type	Dynamic Range	Resolution	Accuracy
Capacitive sensors	10 μm–10 mm	2.4 nm	10 nm–1 μm
Eddy-current sensors	100 μm–80 mm	1 nm	100 nm–10 μm
LVDTs	0.5 mm–500 mm	5 nm	2 μm–1 mm
Linear optical grating	280 mm	1 nm	±140 nm
Laser interferometers	250 mm	0.49 nm	±100 nm
Optical fiber sensors	meters	nanometer	nanometer
MWP sensors	3.5 km	3 μm	3.5 μm

**Table 2:** Optical path difference of the MI with no scanning mirror in the sensing arm.

Path	Optical Path Difference
L1 + L2 + L3 + L6	0
L1 + L4 + L3 + L6	1.2
L1 + L2 + L5 + L6	2.08
L1 + L4 + L5 + L6	3.28
